# Endemic Carbapenem-resistant *Pseudomonas aeruginosa* with Acquired Metallo-β-lactamase Determinants in European Hospital

**DOI:** 10.3201/eid1003.020799

**Published:** 2004-03

**Authors:** Cristina Lagatolla, Enrico A. Tonin, Carlo Monti-Bragadin, Lucilla Dolzani, Francesca Gombac, Claudia Bearzi, Elisabetta Edalucci, Fabrizia Gionechetti, Gian Maria Rossolini

**Affiliations:** * Università di Trieste, Trieste, Italy; † Università di Siena, Siena, Italy

**Keywords:** Pseudomonas aeruginosa, carbapenem-hydrolyzing beta-lactamase, drug resistance, multiple, bacterial, bacterial typing, hospital infection

## Abstract

Acquired metallo-β-lactamases (MBLs) can confer broad-spectrum β-lactam resistance (including carbapenems) not reversible by conventional β-lactamase inhibitors and are emerging resistance determinants of remarkable clinical importance. In 2001, multidrug-resistant *Pseudomonas aeruginosa* carrying *bla*_VIM_ MBL genes were found to be widespread (approximately 20% of all *P. aeruginosa* isolates and 70% of the carbapenem-resistant isolates) at Trieste University Hospital. Clonal diversity and heterogeneity of resistance determinants (either *bla*_VIM-1_-like or *bla*_VIM-2_-like) were detected among MBL producers. This evidence is the first that acquired MBLs can rapidly emerge and establish a condition of endemicity in certain epidemiologic settings.

Bacterial pathogens bearing acquired metallo-β-lactamase (MBL) genes exhibit a broad-spectrum resistance to β-lactams that is not reversible by serine-β-lactamase inhibitors (e.g., clavulanate and penicillanic acid sulphones), since MBLs are capable of hydrolyzing most β-lactams and are not susceptible to inhibitors. Because of the efficient carbapenemase activity of these enzymes, the resistance profile of MBL producers notably includes also carbapenems, which are the β-lactams with the broadest spectrum of activity and are among the “last resort” drugs for the treatment of gram-negative nosocomial infections. In addition, MBL producers most often exhibit resistant phenotype to additional classes of drugs since they originate nosocomially and acquired MBL genes typically cluster with other drug resistance determinants in the variable region of multi-resistance integrons (*1*–*3*). For these reasons, infections caused by MBL producers can pose a substantial challenge for antimicrobial chemotherapy.

The IMP and VIM enzymes are the most common types of acquired MBLs (*2*,*3*). The IMP enzymes were first reported in Japan (*4*), while the VIM enzymes were first reported in Europe (*5*), but both types of enzymes are now emerging in Asia, Europe, and the Americas as acquired resistance determinants in nosocomial isolates of *Enterobacteriaceae*, *Pseudomonas aeruginosa*, *Acinetobacter* spp. and other nonfastidious, gram-negative nonfermenters (*3*). The VIM-1 enzyme is 90% amino acid homologous with the VIM-2 variant and <40% amino acid homologous with the IMP enzymes (*3*). Both types of resistance genes are carried on mobile gene cassettes inserted into plasmid- or chromosomal-borne integrons, a location that eventually facilitates horizontal spreading among different strains (*3*).

Thus far, strains with acquired MBLs have usually been reported sporadically or as causing small nosocomial outbreaks (*4*,*6*–*8*), while longitudinal surveys have demonstrated, at most, a low-level endemicity of MBL producers in hospitals where similar strains have been detected (*9*,*10*). One major hospital outbreak, caused by an MBL-producing *P. aeruginosa* clone, was recently reported in Greece (*11*). We describe the emergence of high-level-endemicity for MBL-producing *P. aeruginosa*, which has recently occurred in a hospital setting of southern Europe.

## The Survey

In the University Hospital of Trieste (northern Italy, at the border with Slovenia), clinical isolates of *P. aeruginosa* producing VIM-type MBLs were detected sporadically, for the first time, in 1999 (*12*). In 2001, a significant increase in the prevalence of imipenem-resistant *P. aeruginosa* isolates was observed at the Laboratory of Clinical Microbiology of that hospital (29%, vs. 19% in 2000 and 21% in 1999, respectively; p < 0.001 according to the χ^2^ test; statistical analyses were conducted with Epi Info statistical software, version 6.03, Centers for Disease Control and Prevention, Atlanta, GA).

Of the 444 nonreplicate imipenem-resistant *P. aeruginosa* isolates collected in 2001, a total of 89 were randomly selected and analyzed for acquired MBL genes of the *bla*_IMP_ and *bla*_VIM_ types in dot-blot hybridization experiments carried out with purified genomic DNA spotted (0.5 μg per spot) on positively charged nylon membranes (ZetaProbe, Bio-Rad, Hercules, CA) with digoxygenin-labeled DNA probes. The probes were polymerase chain reaction amplicons containing internal fragments of the *bla*_IMP-1_ (754–1,114 nt, EMBL/GenBank database entry S71932) or of the *bla*_VIM-1_ gene (3,366–3,888 nt, EMBL/GenBank database entry Y18050), respectively obtained using primers IMP-DIA (forward, 5′-GGAATAGAGTGGCTTAATTCTC; reverse, 5′-GTGATGCGTCYCCAAYTTCACT) and VIM-DIA (forward, 5′-CAGATTGCCGATGGTGTTTGG; reverse, 5′-AGGTGGGCCATTCAGCCAGA) as described previously (*13*). Hybridization was carried out under conditions that allowed recognition, by each probe, of different allelic variants of the corresponding MBL determinant. None of the imipenem-resistant isolates were recognized by the *bla*_IMP_ probe, while 64 (72%) were recognized by the *bla*_VIM_ probe. In the 64 *bla*_VIM_-positive isolates, the nature of the determinant was further investigated by analysis of the *Rsa*I restriction fragment length polymorphism of the gene region amplified by the VIM-DIA primers as described previously. With this approach, the determinant was identified as *bla*_VIM-1_-like in 54 isolates (84%), and as *bla*_VIM-2_-like in the remaining 10 isolates (16%).

The sources of the 64 *bla*_VIM_-positive isolates were 52 inpatients from 15 different wards (including 10 medical wards, 4 surgical wards, and an intensive care unit), 5 patients from 4 different long-term care facilities for elderly persons, and 7 outpatients (Table 1). The degree of genomic relatedness of these isolates was investigated by Random Amplification of Polymorphic DNA (RAPD) (*14*) and by Amplified Fragment Length Polymorphism (AFLP) (*15*). Electrophoretic profiles generated by the techniques described earlier were compared by the GelComparII software (Applied Maths, Kortrijk, Belgium). Consistent results were obtained with both typing methods. Isolates sharing a Dice similarity coefficient >0.88 comparing their RAPD-profiles were assigned to the same cluster. Results of molecular typing indicated that most *bla*_VIM_-positive isolates (61 [95%]) belonged to either of two clusters, indicated as cluster A and B respectively, while the remaining three isolates were unrelated with those clusters and also among each other (Figure). Cluster A included 53 isolates, all containing *bla*_VIM-1_-like determinants. They were widely distributed in the hospital (15 wards), and were also found in three long-term care facilities and in six outpatients. Cluster B included eight isolates, all containing *bla*_VIM-2_-like determinants. The isolates were from four wards where isolates of cluster A had also been detected. Of the three sporadic isolates, one (carrying a *bla*_VIM-2_-like gene) was from a ward where isolates of clusters A and B had also been detected, the second (also carrying a *bla*_VIM-2_-like gene) was from a long-term care facility different from those yielding isolates of cluster A, and the third (carrying a *bla*_VIM-1_-like gene) was from an outpatient (Table 1). Genotyping of the 25 *bla*_VIM_-negative isolates indicated that 5 belonged in cluster A, 1 in cluster B, while the remaining 19 were unrelated to the VIM producers and were overall distributed among 6 different genotypes (Table 1).

**Table 1:** Genetic relatedness, presence of MBL determinants, and distribution of the 89 imipenem-resistant *Pseudomonas aeruginosa* isolates^a^

No. of isolates	RAPD–AFLP genotypes^b^	*bla*_VIM_ allele	Hospital wards (patients)	Long-term care facilities (patients)	Outpatients	
*bla*_VIM_-positive						
53	A	*bla*_VIM-1_-like	15 (43)	3 (4)	6	
8	B	*bla*_VIM-2_-like	4^c^ (8)	-	-	
1	C	*bla*_VIM-2_-like	1^d^ (1)	-	-	
1	D	*bla*_VIM-2_-like	-	1 (1)	-	
1	E	*bla*_VIM-1_-like	-	-	1	
*bla*_VIM_-negative						
5	A	None	2 (3)	-	2	
1	B	None	1 (1)	-	-	
19	F-G-H-I-J-K^e^	None	8 (16)	1 (1)	2	

^a^ MBL, metallo-β-lactamase. ^b^RAPD–AFLP, Random Amplification of Polymorphic DNA–Amplified Fragment Length Polymorphism. Results obtained with the two genotyping techniques were always consistent with each other. ^c^In these wards isolates of cluster A were also detected. ^d^In this ward isolates of clusters A and B were also detected. ^e^Genotypes F to K included a number of isolates ranging from 1 to 7.

**Figure:**
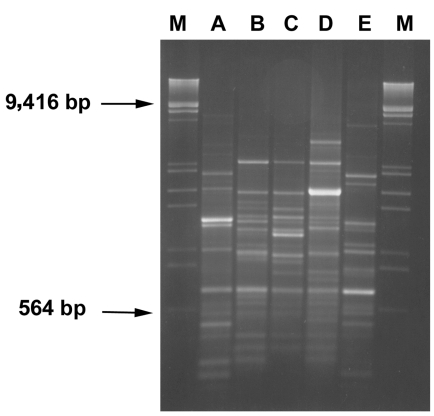
RAPD profiles of *bla_VIM_* positive strains. Amplification products (8 μL) obtained with primer 208 (5′-ACGGCCGACC-3′) (*14*) were run on 2% agarose gel. Lanes A-E: RAPD-types as indicated in Table 1. Lanes M: λDNA digested with *Eco*RI and *Hind*III.

Imipenem MICs for the *bla*_VIM_-positive isolates were always >64 μg/mL (range 64–512 μg/mL), while being always <64 μg/mL for the hybridization-negative isolates. Most of the *bla*_VIM_-positive isolates (49 of 64 [76%]) exhibited a multidrug-resistant phenotype including all the tested drugs (imipenem, meropenem, ceftazidime, piperacillin, aztreonam, amikacin, gentamicin, tobramycin, and ciprofloxacin), except polymixin B. On the other hand, this virtually panresistant phenotype was observed in 7 (28%) of 25 *bla*_VIM_-negative isolates (Table 2).

**Table 2:** Antimicrobial susceptibility of the 89 imipenem-resistant *Pseudomonas aeruginosa* isolates^a^

Drug resistance profile^b^										*bla*_VIM_ status		
										*bla*_VIM-1_ (n = 54) (%)	*bla*_VIM-2_ (n = 10) (%)	*bla*_VIM_-negative (n = 25) (%)
Imi	Mem		Caz	Pip	Atm	Ak	Gm	Tob	Cip	39 (72)	10 (100)	7 (28)
Imi	Mem		Caz	Pip	Atm		Gm	Tob	Cip	11 (20)	-	6 (24)
Imi		Mem	Caz	Pip			Gm	Tob	Cip	1 (2)	-	1 (4)
Imi		Mem	Caz	Pip		Ak	Gm	Tob	Cip	2 (4)	-	-
Imi		Mem	Caz			Ak	Gm	Tob	Cip	1 (2)	-	-
Other^c^										-	-	11 (44)

^a^All isolates were susceptible to polymixin B. The percentage of isolates resistant to all the tested drugs (except polymixin B) was significantly higher among *bla*_VIM_-positive isolates (76 *vs*. 28%; p < 0.001, according to the χ^2^ test). ^b^Imi, imipenem; Mem, meropenem; Caz, ceftazidime; Pip, piperacillin; Atm, aztreonam; Ak, amikacin; Gm, gentamicin; Tob, tobramycin; Cip, ciprofloxacin. ^c^Strains resistant to fewer than 5 antibiotics.

## Conclusions

Our findings are of concern since they demonstrate that acquired MBLs can rapidly emerge and become a major cause of broad-spectrum β-lactam resistance among nosocomial pathogens. In our setting *bla*_VIM_-positive *P. aeruginosa* isolates, which were sporadically detected for the first time in 1999 (*12*), represented approximately 20% of all *P. aeruginosa* isolates and 70% of the carbapenem-resistant *P. aeruginosa* isolates, respectively, during 2001. These figures exceed those reported for MBL producers from other settings (*7*,*9*,*10*). As an additional matter of concern, the *bla*_VIM_-positive isolates were significantly more resistant than the *bla*_VIM_-negative isolates to non-β-lactam antimicrobial agents as well.

In this survey, the *bla*_VIM_-positive isolates were detected on a regular basis during the year and appeared to be widely distributed in the hospital and even outside of it. Molecular characterization showed the simultaneous circulation of different *bla*_VIM_ alleles (either *bla*_VIM1_-like or *bla*_VIM-2_-like) in multiple *P. aeruginosa* clones. Overall, these findings suggest that *bla*_VIM_ determinants have rapidly established a condition of high-level endemicity in this area. To the best of our knowledge, this study is the first in which a similar condition has been reported. Even the large outbreak reported in Greece was caused by a single clone and was apparently confined to the hospital wards (*11*). The finding of *bla*_VIM_-negative *P. aeruginosa* isolates showing the same genotype as that of the two major clusters of *bla*_VIM_-positive strains suggests a likely acquisition of the MBL determinants by strains already endemic in this area, followed by clonal expansion of the *bla*_VIM_-positive strains.

The possibility that spreading transferable MBL genes among nosocomial gram-negative pathogens could emerge as a major problem in the clinical setting underscores the need for systematic surveillance of these resistance determinants. Considering that MBL producers were also isolated from outpatients and from long-term care facility patients, even if all of them showed at least one hospital treatment during the 6 months before, surveillance should not be restricted to nosocomial isolates but should also include isolates from community-acquired infections.
